# Chloroplast Genomes of Two Species of *Cypripedium*: Expanded Genome Size and Proliferation of AT-Biased Repeat Sequences

**DOI:** 10.3389/fpls.2021.609729

**Published:** 2021-02-09

**Authors:** Yan-Yan Guo, Jia-Xing Yang, Hong-Kun Li, Hu-Sheng Zhao

**Affiliations:** College of Plant Protection, Henan Agricultural University, Zhengzhou, China

**Keywords:** plastome expansion, repeat sequence, hybrid assembly, AT-biased base composition, long-read sequencing, palindromic repeat, inversion

## Abstract

The size of the chloroplast genome (plastome) of autotrophic angiosperms is generally conserved. However, the chloroplast genomes of some lineages are greatly expanded, which may render assembling these genomes from short read sequencing data more challenging. Here, we present the sequencing, assembly, and annotation of the chloroplast genomes of *Cypripedium tibeticum* and *Cypripedium subtropicum*. We *de novo* assembled the chloroplast genomes of the two species with a combination of short-read Illumina data and long-read PacBio data. The plastomes of the two species are characterized by expanded genome size, proliferated AT-rich repeat sequences, low GC content and gene density, as well as low substitution rates of the coding genes. The plastomes of *C. tibeticum* (197,815 bp) and *C. subtropicum* (212,668 bp) are substantially larger than those of the three species sequenced in previous studies. The plastome of *C. subtropicum* is the longest one of Orchidaceae to date. Despite the increase in genome size, the gene order and gene number of the plastomes are conserved, with the exception of an ∼75 kb large inversion in the large single copy (LSC) region shared by the two species. The most striking is the record-setting low GC content in *C. subtropicum* (28.2%). Moreover, the plastome expansion of the two species is strongly correlated with the proliferation of AT-biased non-coding regions: the non-coding content of *C. subtropicum* is in excess of 57%. The genus provides a typical example of plastome expansion induced by the expansion of non-coding regions. Considering the pros and cons of different sequencing technologies, we recommend hybrid assembly based on long and short reads applied to the sequencing of plastomes with AT-biased base composition.

## Introduction

The average chloroplast genome (plastome) size of land plants is 151 kb, with most species ranging from 130–170 kb in length, and the average GC content is 36.3% (NCBI database, 4,281 land plant plastomes, March 17, 2020) (Supplementary Table 1). However, previous studies documented that the plastome size of some lineages was extremely enlarged (Chumley et al., 2006; Kim et al., 2015; Blazier et al., 2016; Weng et al., 2017; Lim et al., 2018; Gruzdev et al., 2019; Li H. et al., 2020). The largest chloroplast genome of angiosperm is *Pelargonium transvaalense* (242, 575 bp), with the inverted repeat (IR) region of the species expanding to 87,724 bp (Weng et al., 2017). Of more than 4,000 land plant plastomes from NCBI, 82 sequences examined to date had chloroplast genome sizes over 170 kb, and seven of these species had chloroplast genome sizes over 200 kb (Supplementary Table 1). The expansion of the plastomes of these species is mainly caused by gene duplications in the IR regions (Chumley et al., 2006; Weng et al., 2017; Sinn et al., 2018; Li H. et al., 2020) or the expansion of repeat sequences in non-coding regions (Dugas et al., 2015; Li H. et al., 2020).

Chloroplast genome sequences have been widely used in studies of phylogeny, evolution, and population genetics of angiosperms (Tonti-Filippini et al., 2017). The accurate assembly and annotation of plastome sequences are the foundation of these studies. At present, most of the studies used Illumina short reads for chloroplast genome assembly. However, the short-reads method occasionally does not perform well owing to biased coverage depth, which may lead to fragmented genome assemblies (Ferrarini et al., 2013; Sinn et al., 2018). Recently, a few studies used long reads (Ferrarini et al., 2013; Wu et al., 2014; Chen et al., 2015; Xiang et al., 2016; Cauzsantos et al., 2017; Lee et al., 2020; Zhang et al., 2020) or combined short and long reads for chloroplast genome assembly (Ruhlman et al., 2017; Lin et al., 2018; Wang et al., 2018; Yan et al., 2019; Li H. et al., 2020). Ruhlman et al. (2017) combined long and short read data to investigate repeat sequences in *Monsonia emarginata* (Geraniaceae). Wang et al. (2018) compared short-read (Illumina) data only assembly, long-read (Oxford nanopore) data only assembly, and hybrid assembly involving short- and long-read data to test the accuracy of chloroplast genome assembly. They suggested that hybrid assembly provides highly accurate and complete chloroplast genome assembly.

*Cypripedium* is a genus of Orchidaceae, mainly distributed in the temperate regions of the Northern Hemisphere, with high ornamental and economic values. The genus is a good example to study the evolution of orchids. Owing to the large genome size (average 1C = 31.3 pg) (Leitch et al., 2009), molecular evolution studies of the genus are relatively rare. *Cypripedium* is one of the genera with chloroplast genome expansion (>170 kb) (Supplementary Table 1). At present, three chloroplast genomes of the genus have bene reported: *Cypripedium japonicum* (Kim et al., 2015), *C. formosanum* (Lin et al., 2015), and *C. calceolus* (Zhang et al., 2019). The sequenced chloroplast genomes showed that the genome sizes of the three species are larger than those of most other species of angiosperms, with relatively low GC contents (33.9–34.5%), and that the expansion of the genome size correlated with the expansion of the LSC region. Lin et al. (2015) found an ∼62 kb inversion in *C. formosanum*. However, the inversion was not observed in *C. japonicum*, which is the sister species of *C. formosanum*. In addition, Kim et al. (2015) found AT-rich regions in *C. japonicum*, and owing to the difficulty of sequencing poly(A), poly(T), or poly(AT) regions, they proposed a method for improving the success rates of these AT-rich regions, but the method was limited. Considering that the extents of chloroplast genome expansion and genome structure variation at the genus level are unknown, *Cypripedium* provides an ideal example to study the chloroplast genome evolution of angiosperm with expanded plastome size.

In this study, we selected two species of *Cypripedium* with clear morphological and habitat differentiation to investigate the chloroplast genome variation in the genus. *Cypripedium tibeticum* is widely distributed in southwest China, and *Cypripedium subtropicum* is restricted to southeast Yunnan Province and northern Vietnam. We sequenced, assembled, and annotated the complete chloroplast genomes of *C. tibeticum* and *C. subtropicum* using a combination of Illumina and PacBio sequencing platforms; then, we compared the two genomes with the previously reported chloroplast genomes of the genus in terms of genome size, gene number, genome structure, GC content, and substitution rates of the coding genes; finally, we discussed the expansion mechanism of *Cypripedium* and investigated the repeat sequences of the genus.

## Materials and Methods

### Taxon Sampling and Library Construction

We sampled fresh leaves of *C. tibeticum* and *C. subtropicum* from Huanglong, Sichuan and Malipo, Yunnan, China. Total genomic DNA was isolated using the CTAB method (Doyle and Doyle, 1987). The extracted total genomic DNA was used for library construction with 350 bp and 20 kb insert sizes and then sequenced on MGI2000 (MGI, Shenzhen, China) and PacBio RS-II platforms for the short and long reads, respectively.

### Sequence Assembly and Annotation

The potential chloroplast genome reads were filtered in reference to the three chloroplast genomes of the genus reported in previous studies. Short reads were extracted with a script in NOVOPlasty 3.8.1 (Dierckxsens et al., 2017), and long reads were extracted with BLASR (Chaisson and Tesler, 2012). The hybrid assembly of the chloroplast genomes was performed in SPAdes 3.14.0 based on the filtered reads, with careful error correction and different K-mers (21, 33, 55, and 77) (Bankevich et al., 2012). Then, we used coding genes as seed sequences to test the assembly with NOVOPlasty 3.8.1 (Dierckxsens et al., 2017). The two methods generated almost identical results, except for the AT-biased repeat regions, and the short read only method failed to obtain the assemblies of these regions. The assembled sequences were annotated in Geneious Prime 2020 (Biomatters Ltd., Auckland, New Zealand), coupled with manual correction. The three plastome sequences downloaded from GenBank were reannotated for the following comparison. In addition, we found a paper reporting the chloroplast genome of *C. tibeticum* (GenBank accession No. MN561380) with samples collected from Qinling Mountains of China (Li J. et al., 2020), but the sequence was not yet accessible at the time of the analysis (June 2, 2020), so we were not able to perform further comparisons.

Linear plastome maps were generated with OGDRAW (Greiner et al., 2019). The boundaries of the IR and SC regions were defined by Repeat Finder embedded in Geneious Prime 2020. We calculated GC content in Geneious Prime 2020. Then, we visualized the genome rearrangement of the genus using the progressiveMauve algorithm (Darling et al., 2010) with IRa removed.

### Nucleotide Substitution Rate Analyses

We used the CODEML program in PAML v. 4.9 (model = 0) (Yang, 2007) to calculate the average non-synonymous substitution rate (dN) and synonymous substitution rate (dS) for 79 protein coding genes by the F3X4 codon model. Gapped regions were excluded for rate estimation (cleandata = 1). The input tree [*C*. *subtropicum*, (*C*. *formosanum*, *C*. *japonicum*), and (*C*. *calceolus*, *C*. *tibeticum*)]simplified from Li et al. (2011) was used for the following analyses. In *matK*, only three sequences could be used for substitution rate estimation, and *C*. *formosanum*, (*C*. *calceolus*, *C*. *tibeticum*) was used as input tree. In addition, we counted the numbers of indels in the intergenic spacer regions and introns in the plastomes of *Cypripedium* using DnaSP v6.12.03 (Rozas et al., 2017) with unalignable regions removed using GBlocks (Talavera and Castresana, 2007) with the default settings.

### Repeat Sequence Analysis

Simple sequence repeats (SSRs) (≥10 bp) were detected *via* MISA (Beier et al., 2017), and the minimum thresholds for mono-, di-, tri-, tetra-, penta-, and hexa-nucleotides were set to 10, 5, 4, 3, 3, and 3, respectively. In addition, tandem repeats were identified with Tandem Repeats Finder v4.09 (Benson, 1999) with default parameters, the identity of repeats was set at 90%, and overlapped repeats were removed manually. Dispersed repeats (≥30 bp) and palindromic repeats (≥20 bp) were identified with Vmatch^1^.

## Results

### Plastomes of *Cypripedium tibeticum* and *Cypripedium subtropicum*

**FIGURE 1:**
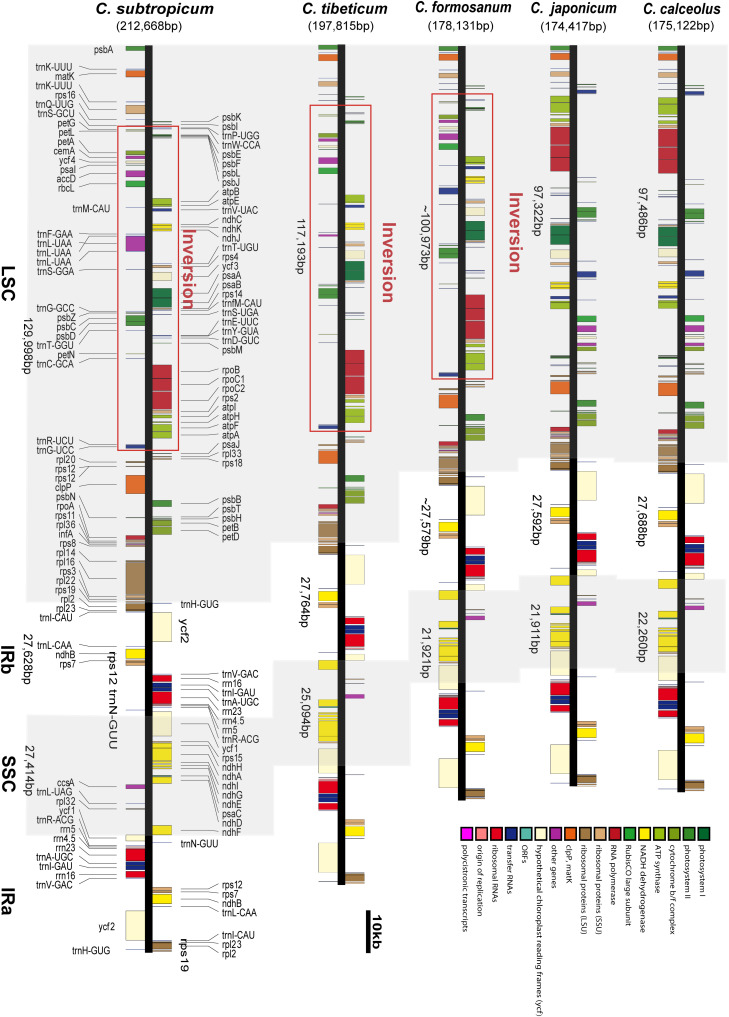
The chloroplast genome structures of five species of *Cypripedium*. The red lines indicate the inversion spanning from *trnG-UCC* to *trnP-UGG* in the LSC region.

**FIGURE 2:**
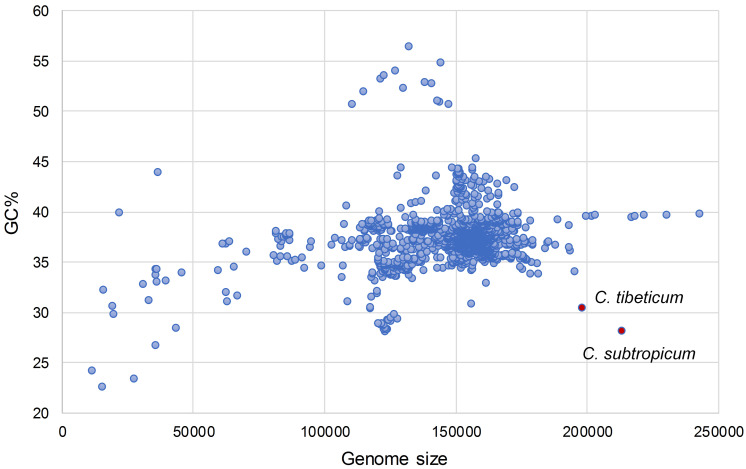
The plastomes of *C. tibeticum* and *C. subtropicum* exhibit atypical size and GC content.

We obtained the full chloroplast genome sequences of 197,815 bp for *C. tibeticum* and 212,668 bp for *C. subtropicum* (GenBank accession Nos. MT937101 and MT937100, respectively). The plastid genomes of the two species showed typical quadripartite structure, with two identical copies of the IR region separated by an LSC region and a small single copy (SSC) region (Figures 1, 2). The LSC regions of the two species expanded to 117,193 and 129,998 bp, respectively, the IR regions of the two species (27,764 and 27,628 bp, respectively) were similar to those of the previously sequenced species, and the SSC regions of the two species (25,094 and 27,414 bp, respectively) were slightly larger than those of the other three species (Table 1). The gene number of the genus was conserved and consisted of 131–132 genes, including 85–86 protein coding genes (seven duplicated in the IR region), 38 tRNAs (eight duplicated in the IR region), and eight rRNAs (four duplicated in the IR region). A total of 15 genes contained one intron, including six tRNA genes (*trnG-UCC*, *trnK-UUU*, *trnL-UAA*, *trnV-UAC*, *trnA-UGC*, and *trnI-GAU*) and nine protein coding genes (*rps16*, *rpl2*, *rpl16*, *rpoC1*, *petB*, *petD*, *atpF*, *ndhA*, and *ndhB*), while the other three protein coding genes (*clpP*, *rps12*, and *ycf3*) contained two introns (Supplementary Table 2). We found pseudogenization of the *matK* gene owing to a single-base deletion-induced frameshift in *C. subtropicum*. Corresponding to the expansion of the genome size and the relatively conserved gene number, the gene density of the genus ranged from 0.62 to 0.75. The GC contents of the total genomes were 30.5% for *C. tibeticum* and 28.2% for *C. subtropicum* (Figure 2), and the GC contents of the two genomes were uneven. The GC contents of the IR regions (42.5 and 42.6%) were higher, whereas the LSC regions (26.5 and 23.7%) and SSC regions (22.4 and 20.6%) had lower GC content (Table 1).

**TABLE 1:** General characteristics of the plastomes of the five *Cypripedium* species included in this study.

Species	*C. subtropicum*	*C. tibeticum*	*C. japonicum*	*C. formosanum*	*C. calceolus*
GenBank No.	MT937100	MT937101	KJ625630*	KJ501998*	MN602053*
Total length (bp)	212,668	197,815	174,417	178,131	175,122
Length of LSC (bp)	129,998	117,193	97,322	∼10,0973	97,486
Length of SSC (bp)	27,414	25,094	21,911	21,921	22,260
Length of IR (bp)	27,628	27,764	27,592	∼27,579	27,688
Number of genes	131 (19)	132(19)	131(19)	132(19)	132(19)
Number of protein coding genes	85 (7)	86 (7)	85 (7)	86 (7)	86 (7)
Number of tRNA genes	38 (8)	38 (8)	38 (8)	38 (8)	38 (8)
Number of rRNA genes	8 (4)	8 (4)	8 (4)	8 (4)	8 (4)
Length of protein coding genes (bp)	77,979	79,626	77,931	79,485	79,680
Length of tRNA genes (bp)	2866	2866	2866	2866	2866
Length of rRNA genes (bp)	9042	9042	9042	9042	9042
Length of coding regions (bp)	89,887	91,534	89,839	91,393	91,588
Length of non-coding regions (bp)	122,781	106,281	84,578	86,738	83,534
Percent of protein coding genes (%)	36.67	40.25	44.68	44.62	45.50
Percent of tRNA genes (%)	1.35	1.45	1.64	1.61	1.64
Percent of rRNA genes (%)	4.25	4.57	5.18	5.08	5.16
Percent of coding regions (%)	42.27	46.27	51.51	51.31	52.30
Percent of non-coding regions (%)	57.73	53.73	48.49	48.69	47.70
Total GC content (%)	28.2	30.5	34.5	33.9	34.4
LSC GC content (%)	23.7	26.5	31.7	30.7	31.6
IR GC content (%)	42.6	42.5	42.7	42.7	42.6
SSC GC content (%)	20.6	22.4	26.4	26.4	26.1
Gene density	0.62	0.67	0.75	0.74	0.75

*Sequences downloaded from GenBank. Numbers in brackets indicate genes duplicated in the IR regions.

Approximately 36.67–45.50% of the genome encoded proteins, 1.35–1.64% encodes tRNA, and 4.25–5.18% encodes ribosomal RNA (Table 1). The length of the coding regions of the five species was approximately 90 kb, whereas the length of non-coding regions ranged from 84 kb in *C. calceolus* to 123 kb in *C. subtropicum* (Table 1). Correspondingly, the non-coding regions of some genes were extremely expanded in the two species; for example, the intron of *rpl16* expanded to 7.5 kb in *C. subtropicum*, the intron of *trnK-UUU* expanded to 7.7 kb in *C. subtropicum* and to 6 kb in *C. tibeticum*, the intergenic region between *psbA* and *trnK-UUU* expanded to 4.3 kb in *C. subtropicum*, the intergenic region between *rbcL* and *atpB* expanded to 4.9 kb in *C. tibeticum*, the intergenic region between *trnL-UAG* and *ccsA* expanded to 2.5 kb in *C. tibeticum* and to 3.4 kb in *C. subtropicum* (Supplementary Table 3). However, some of the non-coding regions were conserved in length, such as the introns of *ndhB* and *rpl2*, which had no length variation (Supplementary Table 3).

**FIGURE 3:**
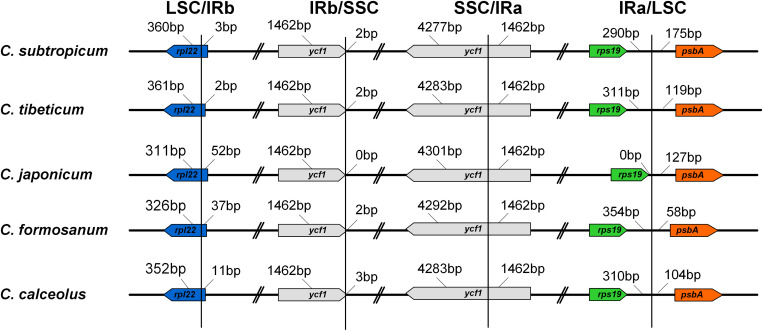
Comparison of the border positions of LSC, SSC, and IR regions among the five *Cypripedium* chloroplast genomes.

The LSC/IR boundary and the IR/SSC boundary were stable in the genus (Figure 3). The LSC/IR_b_ boundary in all five species was located on *rpl22*, while one end of SSC was located on *ycf1*, and the other end of SSC was located on the truncated copy of *ycf1* (Figure 3). The gene order of the genus was conserved, apart from the ∼75 kb inversion spanning from *trnG-UCC* to *trnP-UGG* in the LSC region (Figure 1 and Supplementary Figure 2). In addition, the intergeneric regions adjacent to the long inversions also had high AT contents compared to the other two species without long inversions.

### Repeat Sequences in *Cypripedium*

**FIGURE 4:**
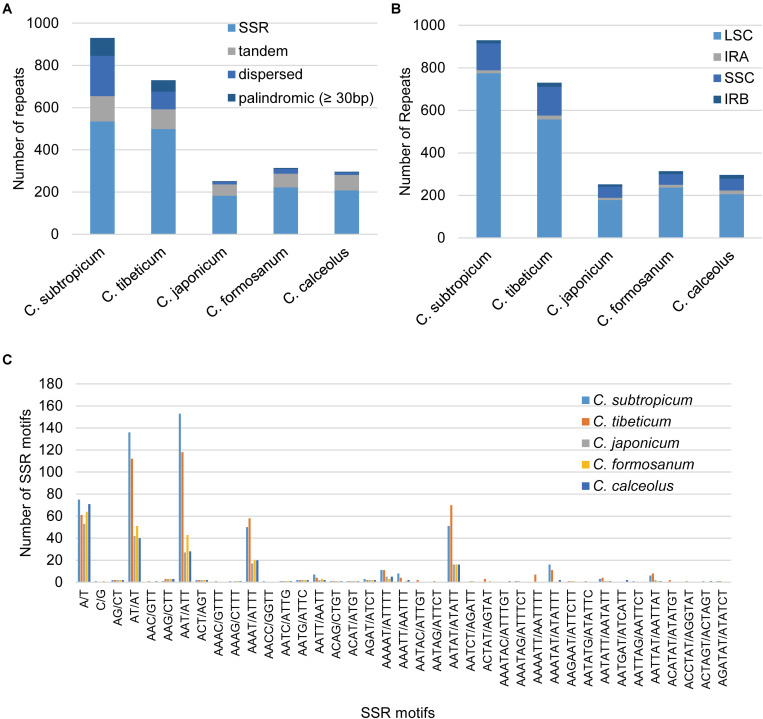
Statistics of the repeat elements in the five *Cypripedium* chloroplast genomes. **(A)** Number and type of repeats; **(B)** the number of repeat sequences in different regions (palindromic repeats excluded); **(C)** number and type of SSR.

The plastomes of *Cypripedium* ranged from 252 repeats (5,362 bp) in *C. japonicum* to 930 repeats (30,453 bp) in *C. subtropicum*, and these repeat sequences represented 3.07% in *C. japonicum* to 14.32% in *C. subtropicum* of the chloroplast genome length, the newly sequenced two species had increased numbers and sizes of repeat sequences (Table 2). The total length of SSR, tandem repeats, and dispersed repeats revealed 3.05-fold, 2.56-fold, and 15.88-fold variations, respectively (Table 2). The repeat sequences in the genus were dominated by SSR (182–535), followed by tandem repeat (54–120) or dispersed repeat (12–191), whereas palindromic repeats were the fewest (three to 84) (≥30 bp) (Table 2). *C. subtropicum* (84) and *C. tibeticum* (54) had more palindromic repeats than the other three species (three to four). All the five species shared a 48-bp (TATAGTGTGGTAGAAAGAGCTATATATAGCTCTTTCTAC CACACTATA) palindromic repeat located in the intergeneric region between *psbM* and *petN*, and most of the other palindromic repeats were species-specific. The longest repeats reached 156 bp in *C. tibeticum* (a 78-bp motif repeated twice), 180 bp in *C. subtropicum* (a 44-bp motif repeated 4.1 times). Unexpectedly, when the repeat length was set to 20 bp, the palindromic repeat number increased substantially (149–5,481) (Table 2), with most of the palindromic repeats having lengths between 20–25 bp (Supplementary Table 5). These repeats were strongly AT-biased, and tandem A/T, AT/TA, AAT/ATT, AAAT/ATTT, and AATAT/ATATT were the five dominant motif types in the SSR (Figure 4). Most of these repeat sequences were located in the non-coding regions of LSC and SSC regions and rarely appeared in the IR region (Figure 4).

**TABLE 2:** Summary of repeat sequences in the plastomes of five *Cypripedium* species.

Species	*C. subtropicum*	*C. tibeticum*	*C. japonicum*	*C. formosanum*	*C. calceolus*
No. of SSRs	535	498	182	222	208
No. of tandem repeats	120	94	54	65	73
No. of dispersed repeats	191	84	12	23	12
No. of palindromic repeats (≥30 bp)	84	54	4	4	3
No. of palindromic repeats (≥20 bp)	5,481	3,008	288	155	149
Total No. of repeats*	930	730	252	314	296
Length of SSR (bp)	6,973	6,753	2,283	2,786	2,638
Length of tandem repeat (bp)	5,250	3,765	2,047	2,654	2,689
Length of dispersed repeat (bp)	13,559	6,127	890	1,638	854
Length of palindromic repeat (≥30bp)	4,671	3,017	142	170	112
Length of repeat sequence (bp)*	30,453	19,662	5,362	7,248	6,293
Percent of repeat sequence*	14.32%	9.94%	3.07%	4.07%	3.59%

*Includes palindromic repeats ≥ 30 bp.

### Nucleotide Substitution Rate Analyses

**FIGURE 5:**
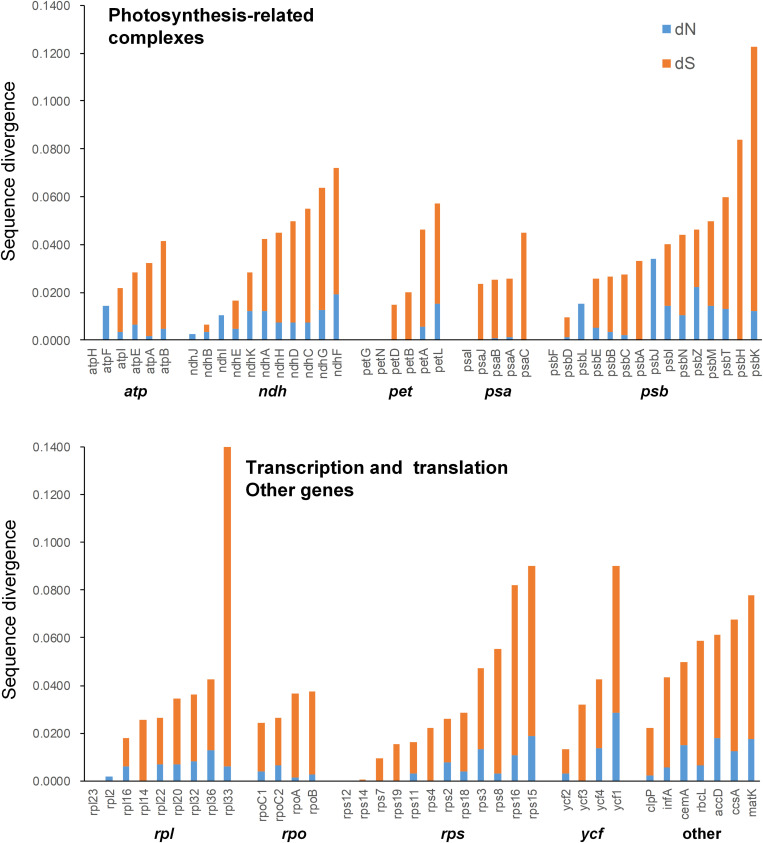
Non-synonymous substitution rate (dN), synonymous substitution rate (dS), and dN/dS for each gene.

Mean synonymous and non-synonymous divergence was extremely low (dN = 0.0069, dS = 0.0287), and varied among genes (Supplementary Table 6). The most rapidly evolving genes in the genus was *rpl33*, while the sequences of some protein coding genes were identical in the five species, such as *atpH*, *petG*, *petN*, *psaI*, *psbF*, *rpl23*, *rps12*, and *rps14* (Supplementary Table 6 and Figure 5). Most of the expanded AT-rich regions were unalignable, and the expanded IGS in LSC and SSC regions had more indels than the non-coding regions without expansion (Supplementary Table 7).

## Discussion

### The Plastome Characters of *Cypripedium tibeticum* and *Cypripedium subtropicum*

To date, the chloroplast genome of *C. subtropicum* (212,668 bp) is the largest of Orchidaceae and the sixth largest of sequenced land plants. Following the expansion of the plastomes, the gene density of the genus decreased to 0.62 in *C. subtropicum* (Table 1), while the gene density of most angiosperms is over 0.80 (the average plastome size of land plants is 151 kb, ∼130 genes, and the gene density is approximately 0.86). The mean synonymous and non-synonymous divergence was low (dN = 0.0069, dS = 0.0287) (Supplementary Table 6). The divergence rate of coding genes is even lower than the coniferous forest tree genus *Picea*, the mean synonymous and non-synonymous of *Picea* was 0.017 ± 0.024 and 0.040 ± 0.031 (Sullivan et al., 2017). The low substitution rates might explain the unresolved relationships among sections in *Cypripedium* (Li et al., 2011).

Interestingly, the overall GC contents of *C. subtropicum* (28.2%) and *C. tibeticum* (30.5%) plastomes are much lower than those of the three species sequenced in previous studies, and the GC content of *C. subtropicum* is the lowest in the sequenced autotrophic species to date (Figure 2 and Table 1). The GC content of the genus is lower than the average GC content of land plant plastomes (37.6%) (Supplementary Table 1). The high AT content is induced by repetitive sequences composed of poly(A), poly(T), or poly(AT) regions in non-coding regions in the single copy region, especially in the LSC region (Kim et al., 2015). Tandem AAT/ATT is the most abundant repeat in newly sequenced species, whereas A/T mononucleotide is the most abundant repeat in the three species sequenced in previous studies (Figure 4). In addition, we found 26 records of the NCBI plastome database with GC content below 30%, and these lower GC content species were restricted to parasitic/mycoheterotrophic plants, mosses, and liverworts (Supplementary Table 5), such as the holoparasitic *Balanophora reflexa* (11.6%) and *B. laxiflora* (12.2%) (Su et al., 2019), the parasitic *Pilostyles hamiltoni* (22.7%) (Bellot and Renner, 2016), and the mycoheterotrophic *Gastrodia elata* (26.7%) (Ma & Jin, unpublished data, MF163256.1). In *Cypripedium*, the GC contents of the coding regions are similar to those of other species, whereas the non-coding regions have relatively lower GC contents, including intergenic spacer regions and introns, and some of the intergenic regions with GC contents lower than 10%, e.g., *trnL-ccsA* (1.3% in *C. subtropicum* to 7.2% in *C. japonicum*) and *psbA-trnK* (2.5% in *C. subtropicum*).

Although the gene order of the two species is conserved, the two species sequenced in this study share an ∼75 kb inversion in the LSC region; the inversion was also found in *C. formosanum* (Lin et al., 2015), and the gene order and orientation in the long inversion were completely conserved (Figure 1). Apart from the long inversion, the chloroplast genome structure of the genus is conserved. According to the section delimitation of the genus (Chen et al., 2013), the three species with long inversions are ascribed to different sections: *C. tibeticum* belongs to section *Cypripedium*, *C. subtropicum* belongs to section *Subtropica*, *C. formosanum* belongs to section *Flabellinervia*, which suggests that the long inversion occurred independently in the genus. Large inversions were also found in other lineages, a 47 kb inversion (*petN* to *clpP*) in *Paphiopedilum fairrieanum* (unpublished data), a 30 kb inversion (*trnG-GCC* to *trnE-UUC*) in *Hevea brasiliensis* (Tangphatsornruang et al., 2011), an 8 kb inversion (*ycf4* to *atpE*) in *Annona cherimola* (Blazier et al., 2016), a 24 kb inversion (*trnQ-UUG* to *trnT-GGU*) in *Viscum minimum* (Petersen et al., 2015), a 42 kb inversion (*clpP* to *trnC-GCA*) in *Passiflora edulis* (Cauzsantos et al., 2017), and a length portion of LSC (*trnH-GUG* to *trnT-GGU*) in *Asarum* (Sinn et al., 2018). The mechanism of these long inversions is mostly unclear, but Sinn et al. (2018) found that the inversion in *Asarum* is flanked by long AT-rich regions, and they proposed that intramolecular recombination induced a long inversion in the genus. The AT-biased repeat sequences were also found in the flanking regions of the three *Cypripedium* species with the long inversion, but there are some other regions of the plastome with AT-rich repeat sequences; thus, the relationship between the inversion and the AT-rich sequences remains uncertain.

Furthermore, despite the increases in the chloroplast genome size of the two sequenced species, the gene number (131–132 genes) of the genus is rather conserved, and the genus encodes all the coding genes commonly found in the chloroplast genomes (Table 1 and Supplementary Table 2). The one gene variation in the two newly sequenced species was due to the pseudogenization of *matK* in *C. subtropicum*. The pseudogenization of *matK* was also observed in *C. japonicum* (Guo et al., 2012; Kim et al., 2015). Interestingly, the pseudogenization of *matK* occurred independently, and both pseudogenization events were frameshift mutations induced by non-triplet nucleotide deletions following a 10-bp mononucleotide (T) repeat 294 bp from the start codon, a 10-bp deletion and a 1-bp deletion (Supplementary Figure 1). The non-triplet indels of *matK* have been reported in previous studies (Kores et al., 2000; Freudenstein and Senyo, 2008; Kocyan et al., 2008; Logacheva et al., 2011), and 82% of the pseudogene entries in GenBank are from Orchidaceae (Barthet et al., 2015). *MatK* is a rapidly evolving chloroplast gene that encodes maturase in the plastome and is related to the splicing of the group IIA introns of seven genes (*atpF*, *rpl2*, *rps12*, *trnV-UAC*, *trnI-GAU*, *trnA-UGC*, and *trnK-UUU*) in land plants (Zoschke et al., 2010). Some angiosperm lineages present coevolution between *matK* and the seven group IIA introns, such as the parasitic *Cuscuta* (McNeal et al., 2009) and the mycoheterotroph *Rhizanthella gardneri* (Delannoy et al., 2011). However, there is no parallel loss of *matK* and the seven group IIA introns in *Cypripedium*: *matK* in *C. subtropicum* and *C. japonicum* are pseudogenes, while the seven group IIA introns are all retained in the two species. We infer that the *matK* gene in the genus might be at the transition from a functional gene to a pseudogene, and that there are other mechanisms regulating the splicing of the group IIA introns.

### The Expansion Mechanism of *Cypripedium*

The plastome expansion of *Cypripedium* is strongly correlated with the proliferation of AT-biased non-coding regions. The five species with larger chloroplast genomes all belong to *Pelargonium* of Geraniaceae (Supplementary Table 1). However, the plastomes of the two genera expanded in different ways. The plastomes of *Pelargonium* incorporated a large portion of what was once the SSC region into the IR region, which induced massive gene duplications (Chumley et al., 2006; Weng et al., 2017). In *Cypripedium*, the lengths of the IR region (27,764 and 27,628 bp) and the SSC region (27,764 and 27,628 bp) are conserved, but the LSC regions of the two species expanded to 117,193 and 129,998 bp respectively, which are 20–30 kb larger than the LSC regions of the three species sequenced in previous studies (Figure 1 and Table 1; Kim et al., 2015; Lin et al., 2015; Zhang et al., 2019). The coding regions of the sequenced species were approximately 90 kb, and the non-coding regions varied in different species. The non-coding region of *C. subtropicum* expanded to 122,781 bp (approximately 57.73% of the chloroplast genome), whereas the non-coding region of *C. japonicum* was 83,534 bp (approximately 47.7% of the chloroplast genome) (Table 1). Given that coding regions of the genus are conserved, we infer that the plastome expansion in the genus is strongly correlated with the proliferation of non-coding regions, especially the non-coding regions in LSC regions, and the two species are typical examples of plastome expansion without gene duplication. These AT-rich repeat sequences led to the plastome expansion of *Cypripedium*. Dispersed repeats contributed most to plastome expansion, followed by SSR and then tandem repeats (Table 2). The expanded regions appeared as unalignable insertions, where the number of indels are correlated with the length of the non-coding regions (Supplementary Table 7). Furthermore, other studies have proposed that repeat sequences lead to plastome expansion; however, the expansion of these species is also associated with gene duplications caused by boundary shifts in IR regions, e.g., watercress (Yan et al., 2019) and *Rhododendron delavayi* (Li H. et al., 2020). Interestingly, short palindromic repeats (20–25 bp) are prevalent in the plastomes of *C. subtropicum* and *C. tibeticum* (Supplementary Table 5). Smith (2020) indicated that panlindromic repeats are mutational hotspots, and contribute to plastome expansion in chlamydomonadalean *Chlorosarcinopsis eremi*. In addition to this study, the proliferation of non-coding regions was mainly documented in algae (Muñoz-Gómez et al., 2017; Gaouda et al., 2018; Smith, 2018), and the non-coding DNA of *Haematococcus lacustris* comprises over 90% of the plastome (Smith, 2018). In addition, we also found high AT regions in *Paphiopedilum* (*trnS-trnG*, *trnE-trnT*, and *trnP-psaJ*) (unpublished data). AT-rich regions were also found in some other genera, e.g., *Asarum* (Sinn et al., 2018). However, the AT-rich regions in most cases do not contribute to plastome size expansion.

Finally, there is coverage bias related to the GC content in the short-read sequencing technologies (Browne et al., 2020), which means that the AT-rich regions exhibit under-coverage in the high-throughput dataset compared to the GC neutral regions. In this study, we failed to obtain parts of the plastomes of the two species through a short-read dataset, especially the AT-rich repetitive regions. We are also unable to circularize the plastomes of some *Paphiopedilum* species due to the lengthy AT-rich regions (unpublished data). Sinn et al. (2018) obtained fragmented plastomes in *Asarum* owing to the lengthy AT-biased regions. Moreover, Zhang et al. (2020) found that two repeat fragments were missing in the short-read assembly compared to the long-read assembly. In contrast, most studies indicated that the coverage depth of long-read sequencing is relatively even (Ferrarini et al., 2013) and could yield highly-accurate plastome assemblies (Wang et al., 2018). Considering the limitation of short-read sequencing and the fact that most of the plastomes in GenBank are obtained based on short-read data, the species with AT-biased plastomes might be underrepresented. Considering the pros and cons of different sequencing technologies, we recommend that the research of plastomes with AT-biased base composition and lengthy repetitive sequence use hybrid assembly, which will take advantage of the high throughput of second-generation sequencing and the longer read length of third-generation sequencing and reduce the coverage biases introduced by DNA sequencing methods, especially the species containing long repetitive elements, and the combination of the two sequencing platforms will greatly simplify the assembly.

## Data Availability Statement

The datasets presented in this study can be found in online repositories. The names of the repository/repositories and accession number(s) can be found in the article/Supplementary Material.

## Author Contributions

Y-YG conceived and designed the study. Y-YG, J-XY, H-SZ, and H-KL analyzed the data. Y-YG wrote the manuscript. All authors contributed to the article and approved the submitted version.

## Conflict of Interest

The authors declare that the research was conducted in the absence of any commercial or financial relationships that could be construed as a potential conflict of interest.

## Footnotes

1 http://www.vmatch.de/

## Supplementary Material

Supplementary Figure 1 Alignment of the *matK* gene of the genus. The red arrows indicate the non-triplet nucleotide deletions.

Supplementary Figure 2 Synteny alignment of chloroplast genomes of *Cypripedium*. Locally collinear blocks of the sequences are color-coded and connected by lines.

Supplementary Table 1 List of land plants with sequenced plastomes available from GenBank. The species with expanded chloroplast genomes (>170 kb) are marked in red.

Supplementary Table 2 List of genes identified in the chloroplast genomes of *Cypripedium.*

Supplementary Table 3 Lengths of intergenic spacer regions and introns in the plastomes of *Cypripedium*.

Supplementary Table 4 Species with GC content lower than 30% from GenBank database.

Supplementary Table 5 The number of palindromic repeats (≥20 bp) in the chloroplast genomes of *Cypripedium.*

Supplementary Table 6 Non-synonymous substitution rate (dN), synonymous substitution rate (dS), and dN/dS for each gene.

Supplementary Table 7 Numbers of indels in intergenic spacer regions and introns in the plastomes of *Cypripedium*.
