# Supplementary figures and images for: Chloroplast Genomes of Two Species of Cypripedium: Expanded Genome Size and Proliferation of AT-Biased Repeat Sequences
Source: Front Plant Sci. 2021 Feb 9;12:609729. doi: 10.3389/fpls.2021.609729 (PMC7900419; doi:10.3389/fpls.2021.609729)

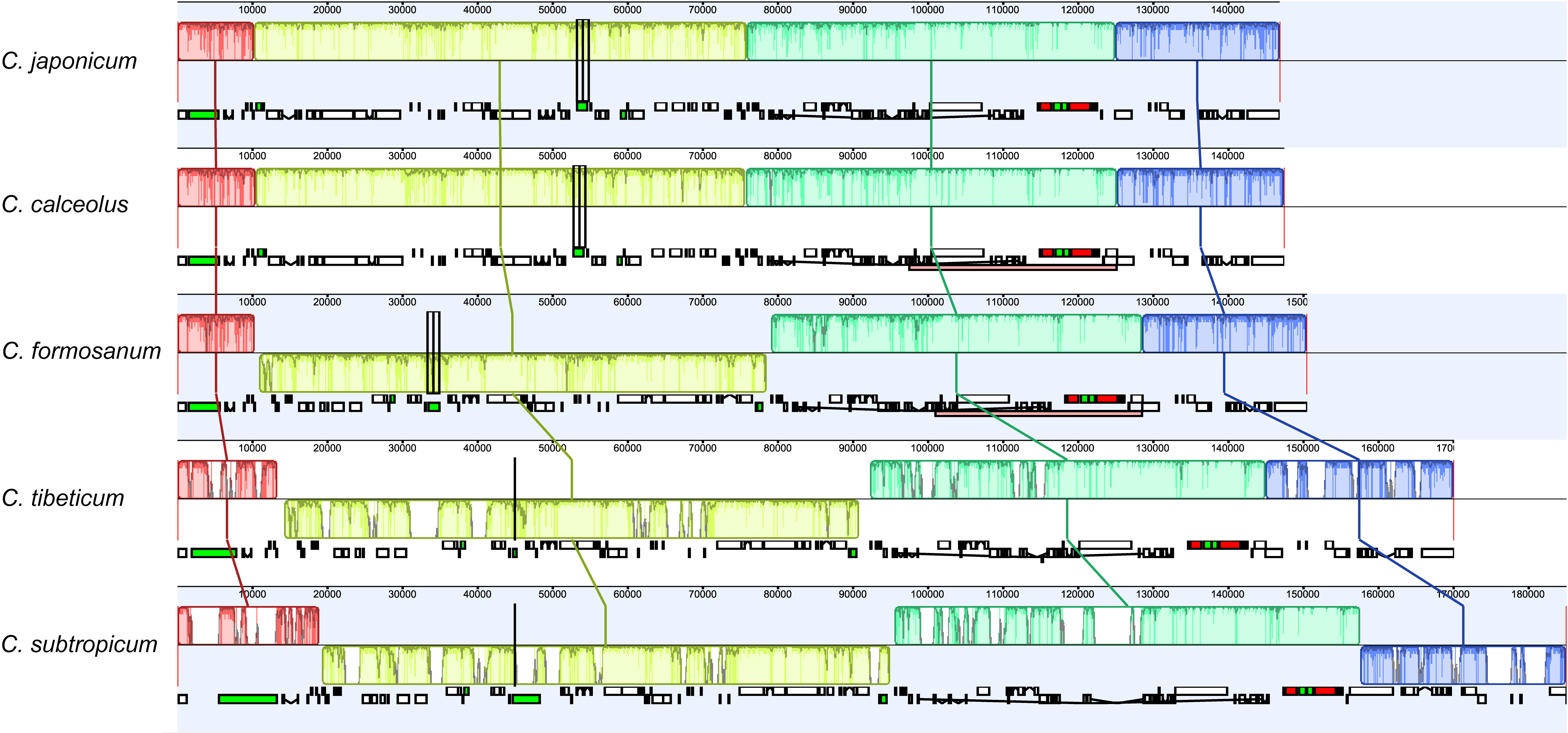

Supplement: Supplementary Figure 2 — Synteny alignment of chloroplast genomes of Cypripedium. Locally collinear blocks of the sequences are color-coded and connected by lines. [file Image_2.TIF]
